# Efficacy of Cross-Linked Hyaluronic Acid Gel for the Reduction of Post-operative Obstructive Symptoms Due to Adhesions

**DOI:** 10.7759/cureus.22469

**Published:** 2022-02-21

**Authors:** Wasim H Khan, Ali Abaid, Usman I Butt, Muhammad U Warraich, Mahmood Ayyaz, Ahsan Shafiq

**Affiliations:** 1 General Surgery, Services Hospital Lahore, Lahore, PAK; 2 General Surgery, Hayyat Memorial Teaching Hospital, Lahore, PAK; 3 Surgical Oncology, Shaukat Khanum Memorial Cancer Hospital and Research Centre, Lahore, PAK

**Keywords:** obstructive symptoms, laparotomy, peritonitis, small bowel perforation, hyaluronic acid gel

## Abstract

Background

Adhesions occur frequently after surgery. A number of methods are being employed for reducing post-operative adhesions. The purpose of this study was to determine the efficacy of hyaluronic acid gel in the reduction of post-operative bowel obstruction symptoms in patients undergoing emergency laparotomy due to small bowel perforation and presenting with peritonitis.

Methods

In this experimental study, 78 patients were evaluated. All had presented to the emergency department with peritonitis secondary to small bowel perforation. Exploratory laparotomy was performed and after thorough lavage, a loop stoma was formed on the right side of the abdomen with an injection of hyaluronic acid gel injected into the abdominal cavity before the closure of the abdomen. Postoperative obstructive symptoms at one, three, and six months were evaluated. All patients underwent stoma reversal at three months.

Results

Obstructive bowel symptoms were seen in 18 patients (23.07%) patients. The cumulative incidence of obstructive symptoms at three months was only 8.97% but after the second intervention without protective gel, it increased to 23.07% at six months.

Conclusions

Cross-linked hyaluronic acid gel was effective in the reduction of post-operative bowel obstructive symptoms due to adhesions in patients who had presented with peritonitis due to small bowel perforation and had undergone exploratory laparotomy with stoma formation.

## Introduction

Surgical adhesions are fibrotic bands that form between various structures of the abdominal cavity usually as a result of tissue trauma. Post-surgical adhesions may be thin or thick fibrous. It is believed that injury to the peritoneal tissue is the usual initiating factor for these. Injury may be in the form of surgery, inflammation, infection, radiation, or a foreign body. However, the exact cause is unknown [1].

Despite the advancements in medical technology adhesions remain a common complication occurring in 50-90 % of surgical operations [2]. Millions of patients get affected annually [3]. Patients without previous surgery are found to have inflammatory adhesions in 10 % of the population while postoperative adhesions have been reported in up to 90% of the patients after surgery. A plethora of studies have documented adhesions after surgery [4-7] and even after laparoscopic surgery [8]. The nature of adhesions depends upon the type of surgery. A number of problems including pain, organ dysfunction, redo surgery, infertility and intestinal obstruction have been attributed to adhesions. Adhesions significantly increase the risk of hemorrhage, iatrogenic perforation, decreased view, and increased operative times during redo surgery. Thus they are a major burden on the individual patient as well as the health care system [9]. The exact cause of adhesion formation post-surgery is still not exactly known, thus, no definitive mechanisms to reduce adhesion formation have yet been developed [10].

Considering the effects and complications of adhesions on both the patients and healthcare system considerable efforts are being made to decrease or stop the formation of adhesions after surgery. Biomaterials that are directly applied to the tissues are being used with varying success. The role of nanoparticles and genetic therapies is also being assessed. A large number of pharmaceutical agents have been used and accessed for the prevention of adhesions. Some have shown a positive effect in animal studies. However, no conclusive data has been found regarding their efficacy. Furthermore, they also have a number of drawbacks related to systemic action and other adverse effects [11]. This has thus led to an increased interest in the prevention of adhesions by the use of barriers. Barriers prevent direct physical contact between two surfaces and thus prevent adhesion formation. A number of barriers are being evaluated such as polytetrafluoroethylene (PTFE), polylactic acid (PLA), polyethylene glycol (PEG), PLA-PEG, hyaluronic acid (HA), alginate (ALG), cellulose (oxidized regenerated) (ORC), carboxymethyl cellulose (CMC), and Icodextrin. However, introduction in clinical practice has been slow due to a number of reasons such as cost, lack of human studies, difficulty in application and preparation [12].

Hyaluronic acid is found in the soft tissues of all vertebrates. It is a glycosaminoglycan present in synovial fluid, vitreous humor, blood vessel walls, the umbilical cord, and connective tissues. It forms a thick solution that tends to cover the areas to which it is applied. As a result, it offers protection against the destruction of the serosa and minimizes tissue injury by minimizing the release of enzymes and oxygen radicals from activated white blood cells of the peritoneum. Various pharmaceutical compositions of hyaluronic acid are used to prevent the formation of postsurgical scars, adhesions, and keloid formation in connection with various surgical procedures. Animal and human studies have shown that hyaluronic acid offers a reduction of postoperative adhesions [11].

Although the use of hyaluronic acid for reducing post-operative adhesions has been studied in a number of elective surgeries, only a few studies have assessed the efficacy of hyaluronic acid in peritonitis. The objective of the study was to determine the efficacy of cross-linked hyaluronic acid gel in the reduction of post-operative bowel obstruction symptoms within six months of surgery.

## Materials and methods

Between July 2019 and July 2021 patients who underwent stoma formation in the emergency department due to small bowel perforation were enrolled in this study. A single-center experimental study was conducted at our hospital. Patients younger than 12 years and older than 70 years, vitally unstable, having a history of previous abdominal surgery or peritoneal dialysis, refusing to participate in the study were excluded from the study. Written informed consent was obtained from all patients before surgery. Approval from the Institutional Ethical Committee (IRB/2019/566/SIMS) was taken prior to the start of the study.

Patients who had presented with acute abdomen were included in the study. Exploratory laparotomy was performed in all patients. Any patients with etiology other than small bowel perforation were excluded from the study. After thorough lavage loop stoma was formed on the right side of the abdomen. The cross-linked hyaluronic acid gel was injected into the abdominal cavity before the closure of the abdomen. Abdominal closure was done with 1-0 polypropylene sutures. The skin was closed over a corrugated rubber drain with 2-0 polypropylene sutures. A pelvic drain was placed in all cases but kept clamped for the first six hours post-operatively. The same post-operative management was done in all patients. Patient demographic information including age, gender, and BMI was noted. All patients were shifted to wards for post-operative care till discharge. Follow-up was done postoperatively at defined time intervals (one, three, and six months). At follow-up, patients were questioned regarding clinical signs and symptoms suggestive of bowel obstruction. The responses were recorded. Physical examination was done and documented. Patients were also advised to come to the hospital if they had unresolved bowel obstruction symptoms where they received intravenous fluid therapy and broad-spectrum antibiotics as indicated. A nasogastric tube was also placed if small intestinal dilatation was seen on plain abdominal radiographs.

Bowel obstruction was considered to be present if the patient reported the presence of at least three symptoms out of the following: nausea, vomiting, cramping abdominal pain, abdominal distension, and the absence of defecation or flatus for more than 24 hours. Physical examination was done in all such patients (supportive findings included: abdominal tenderness, accentuation of bowel sounds, and tympanic sounds on percussion). The presence of bowel sounds was determined to rule out ileus. We made use of just a plain abdominal X-ray to help in the confirmation and management of the diagnosis. The use of similar criteria was also made in the studies by Kim et al. [13]. Symptoms of bowel obstruction if present within the study period were considered to be due to post-operative adhesion formation.

The endpoint of the study was to determine the incidence of bowel obstruction symptoms six months after primary surgery. Comparison with previously documented incidence was also done. We calculated a sample size of 78 by keeping the confidence interval equal to 95%, margin of error equal to 10%, and the presence of efficacy after hyaluronic acid as 72.3% of the cases in previous studies [14]. The quantitative variables like age, BMI, duration of surgery, and duration of hospital stay were presented by calculating mean and standard deviation. Frequency and percentages were collected for qualitative data like gender, and outcome variable i.e. efficacy as yes or no. Effect modifiers were stratified against age, gender, BMI, duration of surgery, and duration of hospital stay. The post-stratification Chi-square test was applied and P was calculated where a P value equal to or less than 0.05 was taken as significant. All analyses were carried out with SPSS version 22 (IBM Corp., Armonk, NY) software.

## Results

The age range in this study was from 12 to 70 years with a mean age of 41.26 ± 10.53 years. The majority of the patients (51, 65.38%) were between 12 to 45 years of age as shown in Table 1. Out of the 78 patients, 46 (58.97%) were male and 32 (41.03%) were females with a male to female ratio of 1.4:1. The mean BMI was 28.99 ± 3.11 kg/m^2^. The mean duration of surgery was 57.74 ± 10.95 minutes. The mean duration of hospital stay was 4.22 ± 1.44 days. In our study obstructive bowel symptoms were seen in 18 patients (23.07%). 

**Table 1:** General Characteristics

Variables	Mean
Age (in years)	41.26 ± 10.528
Hospital Stay (in Days)	4.22 ± 1.438
BMI (kg/m^2^)	28.99 ± 3.106
Duration of Surgery (minutes)	57.74 ± 10.94
Gender (Male and Female)	46 (58.98%) and 32 (41.02%)

Stratification of efficacy with respect to various variables is shown in Table 2 along with the value of P. All the patients settled with conservative management. The cumulative incidence of obstructive symptoms at three months was only 8.97% but after the second intervention, it increased to 23.07% at six months as shown in Table 3.

**Table 2:** Stratification of efficacy with respect to variables

Variables	Efficacy		P value
	Yes	No	
Age ( in years)			
12-45	39	12	0.896
46-70	21	06	
Gender			
Male	38	08	0.153
Female	22	10	
Duration of surgery( in minutes)			
< 60	38	12	0.796
>60	22	06	

**Table 3:** Incidence of obstructive symptoms at 1, 3, and 6 months

	1 month	3 months	6 months
Obstructive Symptoms	2 (2.56%)	5 (6.41%)	11 (14.10%)

## Discussion

In this study, we found the incidence of bowel obstruction symptoms to be 23.07% within six months after exploratory laparotomy. The cumulative incidence of bowel obstruction was 2.56% at one month, 8.97% at three months, and 23.07% at six months. Although comparable to that documented in previous studies (11.7%-38.5%) this incidence may be due to the fact that the cases included in the study were prone to adhesion formation being post-operative and post peritonitis. To the best of our knowledge, this is the first study to evaluate the effect of hyaluronic acid barrier gel on the development of intraabdominal adhesions in the acute setting secondary to small bowel perforation.

A study done by Carta et al. [15] in patients undergoing gynecological abdominal surgery showed that only 18 % of patients developed adhesions with the use of hyaluronic acid while a systemic review done by Zheng et al. [14] showed that hyaluronic acid preparations could decrease abdominal adhesion after general surgery but had no effect on post-operative intestinal obstruction.

Seventy patients were recruited by Tang et al. for a prospective randomized trial involving rectal resection with ileostomy formation [16]. Adhesion formation and stomal complications at three weeks were the measured outcomes. A significant reduction of the mean adhesion score was seen in the hyaluronic acid group (5.81 ± .5 vs 7.82 ± .6; P=.05). Similarly, Vrijland et al. carried out a prospective multicenter study in which 71 patients underwent Hartmann’s resection [17]. The patients were randomized into hyaluronic acid and control groups. The hyaluronic acid group showed a significant reduction in the severity of adhesions (odds ratio {OR}, .34; 95% CI, .06 -1.98) although no difference in the incidence of adhesions was seen. 

Cohen et al. performed a randomized prospective multicenter trial with 120 patients who were undergoing colectomy and ileal pouch surgery [18]. The patients were randomized into a hyaluronic acid and a control group. Assessment for adhesions was carried out via laparoscope eight to 12 weeks later at ileostomy closure. the hyaluronic acid group was found to have significantly reduced incidence and severity of adhesions. (OR, .23; 95% CI, .08 -.62). Kusunoki et al. randomized 62 patients undergoing rectal cancer surgery into two groups with one group receiving intra-abdominal hyaluronic acid while no intervention was done in the other group [19]. There was a reduction in adhesions in the hyaluronic acid group which was associated with decreased surgical time, less blood loss, and the smaller incision at ileostomy closure. A significant reduction in the incidence of adhesions (OR, .15; 95% CI, .05-.43; P=.001) and the extent of adhesion (mean difference, -25.9%; 95% CI, -40.56 to -11.26; P=.001) was seen in meta-analysis carried out by Kumar et al. [20] in patients undergoing non-gynecologic surgery and similar findings were also noted in the systematic review by Robb and Mariette [21].

We carried out our study in patients who presented with ileal perforation, mostly typhoid. Our study has a number of limitations. Our study is a small experimental study with 78 patients. All of our patients underwent exploratory laparotomy with stoma formation followed by stoma reversal at three months. Stoma reversal was done through the ostomy site. However hyaluronic gel was only inserted at the first operation. The cumulative incidence of obstructive symptoms at three months was only 8.97% but after the second intervention without protective gel, it increased to 23.07% at six months (Table 3). Although we are not sure why but it seems that the incidence of obstructive bowel symptoms increased after the second surgery. Similar findings were also reported by Isa and Bodnar [22]. Our study is a single-center study. Ours is one of the first studies carried out in this part of the world. Furthermore very few studies have evaluated the role of hyaluronic acid in the prevention of adhesions in patients with perforation who are already at high risk of adhesion formation. A recent meta-analysis carried out by Guo et al. showed that hyaluronic acid reduced post-operative bowel obstruction but all included studies involved elective cases [23].

This study showed that cross-linked hyaluronic acid gel treatment after laparotomy results in a reduction in post-operative obstructive symptoms. However, further high-powered randomized studies are required to further elaborate the efficacy of hyaluronic acid gel.

## Conclusions

As per our results cross-linked hyaluronic acid gel was found to be effective in the reduction of post-operative bowel obstructive symptoms due to adhesions. 

## References

[1] Tabibian N, Swehli E, Boyd A, Umbreen A, Tabibian JH (2017). Abdominal adhesions: a practical review of an often overlooked entity. Ann Med Surg (Lond).

[2] Fischer A, Koopmans T, Ramesh P (2020). Post-surgical adhesions are triggered by calcium-dependent membrane bridges between mesothelial surfaces. Nat Commun.

[3] Arung W, Meurisse M, Detry O (2011). Pathophysiology and prevention of postoperative peritoneal adhesions. World J Gastroenterol.

[4] Beck DE, Opelka FG, Bailey HR, Rauh SM, Pashos CL (1999). Incidence of small-bowel obstruction and adhesiolysis after open colorectal and general surgery. Dis Colon Rectum.

[5] Menzies D, Ellis H (1990). Intestinal obstruction from adhesions--how big is the problem?. Ann R Coll Surg Engl.

[6] Nieuwenhuijzen M, Reijnen MM, Kuijpers JH, van Goor H (1998). Small bowel obstruction after total or subtotal colectomy: a 10-year retrospective review. Br J Surg.

[7] Weibel MA, Majno G (1973). Peritoneal adhesions and their relation to abdominal surgery. A postmortem study. Am J Surg.

[8] Kavic SM, Kavic SM (2002). Adhesions and adhesiolysis: the role of laparoscopy. JSLS.

[9] van Goor H (2007). Consequences and complications of peritoneal adhesions. Colorectal Dis.

[10] Capella-Monsonís H, Kearns S, Kelly J, Zeugolis DI (2019). Battling adhesions: from understanding to prevention. BMC Biomed Eng.

[11] Fatehi Hassanabad A, Zarzycki AN, Jeon K, Dundas JA, Vasanthan V, Deniset JF, Fedak PW (2021). Prevention of post-operative adhesions: a comprehensive review of present and emerging strategies. Biomolecules.

[12] Krielen P, Grutters JP, Strik C, Ten Broek RP, van Goor H, Stommel MW (2019). Cost-effectiveness of the prevention of adhesions and adhesive small bowel obstruction after colorectal surgery with adhesion barriers: a modelling study. World J Emerg Surg.

[13] Kim SG, Song KY, Lee HH (2019). Efficacy of an antiadhesive agent for the prevention of intra-abdominal adhesions after radical gastrectomy: a prospective randomized, multicenter trial. Medicine (Baltimore).

[14] Zeng Q, Yu Z, You J, Zhang Q (2007). Efficacy and safety of Seprafilm for preventing postoperative abdominal adhesion: systematic review and meta-analysis. World J Surg.

[15] Carta G, Cerrone L, Iovenitti P (2004). Postoperative adhesion prevention in gynecologic surgery with hyaluronic acid. Clin Exp Obstet Gynecol.

[16] Tang CL, Seow-Choen F, Fook-Chong S, Eu KW (2003). Bioresorbable adhesion barrier facilitates early closure of the defunctioning ileostomy after rectal excision: a prospective, randomized trial. Dis Colon Rectum.

[17] Vrijland WW, Tseng LN, Eijkman HJ (2002). Fewer intraperitoneal adhesions with use of hyaluronic acid-carboxymethylcellulose membrane: a randomized clinical trial. Ann Surg.

[18] Cohen Z, Senagore AJ, Dayton MT (2005). Prevention of postoperative abdominal adhesions by a novel, glycerol/sodium hyaluronate/carboxymethylcellulose-based bioresorbable membrane: a prospective, randomized, evaluator-blinded multicenter study. Dis Colon Rectum.

[19] Kusunoki M, Ikeuchi H, Yanagi H (2005). Bioresorbable hyaluronate-carboxymethylcellulose membrane (Seprafilm) in surgery for rectal carcinoma: a prospective randomized clinical trial. Surg Today.

[20] Kumar S, Wong PF, Leaper DJ (2009). Intra-peritoneal prophylactic agents for preventing adhesions and adhesive intestinal obstruction after non-gynaecological abdominal surgery. Cochrane Database Syst Rev.

[21] Robb WB, Mariette C (2014). Strategies in the prevention of the formation of postoperative adhesions in digestive surgery: a systematic review of the literature. Dis Colon Rectum.

[22] Isa MA, Bodnar OB (2017). Hyaluronic acid solution as a treatment of adhesive intestinal obstruction in children - a positive effect: PS230. Porto Biomed J.

[23] Guo Y, Zhu Q, Chen S (2021). Effect of sodium hyaluronate-arboxycellulose membrane (Seprafilm®) on postoperative small bowel obstruction: A meta-analysis. Surgery.

